# Esketamine Alleviates Neuropathic Pain and Inflammation via miR‐153–3p/AKT3 in CCI Rats

**DOI:** 10.1155/prm/5536335

**Published:** 2026-08-17

**Authors:** Xiumei Zhu, Yawen Zhang

**Affiliations:** ^1^ Department of Anesthesiology, Guannan County First People’s Hospital, Lianyungang 223500, China; ^2^ Department of Neurology, The Third Hospital of Hebei Medical University, Shijiazhuang 050051, China, hebmu.edu.cn

**Keywords:** esketamine, inflammation, miR-153–3p, neuropathic pain

## Abstract

**Background:**

The analgesic effects of esketamine have been reported. MiR‐153–3p is a central player in multiple pathologies of neural injury. The potential interplay between them remains unclear.

**Objective:**

This study investigated whether the analgesic effects of esketamine in neuropathic pain were mechanistically linked to miR‐153–3p.

**Materials and Methods:**

Neuropathic pain was induced in rats through chronic constriction injury (CCI) of the sciatic nerve, followed by esketamine administration. Pain hypersensitivity was assessed by dynamic changes in paw withdrawal mechanical threshold (PWMT) and paw withdrawal thermal latency (PWTL). MiR‐153–3p expression was manipulated via intrathecal injection of lentiviral‐delivered miR‐153–3p mimic. RT‐qPCR was employed for analysis of miR‐153–3p expression and AKT3 mRNA levels. Proinflammatory cytokine levels were quantified by ELISA, while protein expression was detected using Western blot. The interaction between miR‐153–3p and AKT3 was verified by RIP and luciferase reporter assays.

**Results:**

Esketamine alleviated CCI‐induced neuropathic pain by elevating PWMT and PWTL. Esketamine also reduced proinflammatory cytokine release. MiR‐153–3p was significantly reduced in CCI rats, and esketamine administration effectively reversed its decline. MiR‐153–3p upregulation suppressed pain hypersensitivity and proinflammatory cytokines. AKT3 was a target of miR‐153–3p. AKT3 knockdown abolished the effect of MiR‐153–3p inhibition on esketamine protection.

**Conclusion:**

Esketamine mitigated neuropathic pain and reduced proinflammatory cytokine release through the miR‐153–3p/AKT3 pathway.

## 1. Introduction

Neuropathic pain refers to a persistent pain initiated by primary lesions or disease of the somatic sensory nervous system [1], primarily attributable to abnormal nerve function or damage to nerve tissues [2]. This pain originates from the central or peripheral nervous system [3] and frequently manifests as spontaneous pain and heightened pain sensitivity [4]. As a prevalent long‐term complication following various surgical procedures, neuropathic pain is estimated to affect 7%–10% of individuals in the general population [5]. Existing therapies offer only mild‐to‐moderate efficacy, and long‐term use often leads to tolerance and a progressive decline in therapeutic response [6]. Thus, its management remains a major clinical challenge [7].

Esketamine has been widely recognized as a promising therapeutic agent for neuropathic pain [8, 9]. Accumulating evidence has revealed that esketamine exerts multidimensional analgesic effects by targeting neuroinflammation, synaptic plasticity, and glial cell regulation. Studies demonstrate that esketamine alleviates neuropathic pain by suppressing spinal microglial overactivation and migration following nerve injury [10]. In a spared nerve injury rat model, low‐dose esketamine can relieve pain‐induced depressive‐like behaviors via the mGluR5/Homer1a pathway [11]. In chemotherapy‐induced neuropathic pain and anxiety models, esketamine reduced mechanical allodynia through BDNF/TrkB activation [12]. In addition, it modulates astrocytic activity and neuronal excitability to reverse pain hypersensitivity in spinal nerve–ligated mice [13]. Clinically, esketamine has successfully treated refractory cancer–related neuropathic pain, improving quality of life in patients unresponsive to conventional therapies [14]. These findings underscore its promise as a multimodal agent for neuropathic pain.

MicroRNAs (miRNAs) are critical posttranscriptional regulators. MiR‐153–3p has recently emerged as a pivotal player in neuroinflammatory conditions. MiR‐153–3p exhibits direct relevance to nerve injury and pain pathogenesis. Studies have shown that increased miR‐153–3p levels correlate with reduced Alzheimer’s disease susceptibility [15], while its downregulation exacerbates cerebral ischemia/reperfusion injury [16]. Research on cerebral infarction links its expression to neurorepair and inflammation suppression [17]. Notably, in a lipopolysaccharide‐induced neuroinflammation model, miR‐153–3p exerts anti‐inflammatory and antineuronal apoptosis effects by targeting NeuroD1 [18]. Given that neuroinflammation is a central driver of neuropathic pain, miR‐153–3p represents a highly promising target in pain research. However, the expression patterns, signaling pathways, and therapeutic effects of miR‐153–3p in models of neuropathic pain remain entirely unknown. To date, no studies have investigated whether it plays any functional role in the pathogenesis of neuropathic pain. This constitutes a significant knowledge gap.

Importantly, it has been demonstrated that esketamine exerts some of its protective effects by modulating the expression of miRNAs. In a rat model of chronic obstructive pulmonary disease, esketamine modulated the expression of miR‐143–3p and miR‐130a‐3p to alleviate cellular stress and pulmonary inflammation [19]. In mice subjected to chronic restraint stress, it suppresses the overexpression of miR‐132–5p, thereby maintaining synaptic plasticity [20]. In a perioperative neurocognitive disorder model, esketamine modulated mmu‐miR‐155–5p and mmu‐miR‐1a‐3p to regulate neuronal function and synaptogenesis [21]. Although esketamine can regulate various miRNAs in different neurological situations, whether it directly or indirectly regulates miR‐153–3p and whether this regulation contributes to its analgesic effect in neuropathic pain have not yet been studied. Given that neuroinflammation is a major driver of neuropathic pain, and that previous studies have shown that miR‐153–3p exerts anti‐inflammatory effects by targeting NeuroD1 and other inflammatory mediators, we hypothesize that esketamine alleviates neuropathic pain, at least in part, by regulating the expression of miR‐153–3p, thereby inhibiting the neuroinflammatory cascade.

Therefore, we hypothesize that esketamine may alleviate neuropathic pain by modulating miR‐153–3p and its downstream inflammatory pathways. Specifically, esketamine might upregulate miR‐153–3p, which in turn inhibits proinflammatory factors or glial activation, ultimately reducing pain hypersensitivity. This study employed an animal model to characterize the expression pattern of miR‐153–3p and investigate whether the analgesic effects of esketamine were mechanistically linked to miR‐153–3p in neuropathic pain. By addressing these critical gaps, we seek to identify a novel mechanistic insight and a potential therapeutic target for pain intervention.

## 2. Materials and Methods

### 2.1. Experimental Animals

Adult male Sprague–Dawley rats (200–240 g) were obtained from the Shanghai SLAC Laboratory Animal Co., Ltd. The animals were maintained in a controlled environment at 22 ± 1°C and 50% relative humidity, under a 12‐h light/dark cycle. Libitum access to food and water was also provided. All experimental procedures were in strict accordance with the Guide for the Care and Use of Laboratory Animals (2011 edition) [22]. Before experiments, rats were allowed a 1‐week acclimatization period.

This experiment was conducted with the approval of the Animal Ethics Committee of the GuannanCounty First People’s Hospital. All animal experiments in this study were in strict accordance with the protocols stated in the Guide for the Care and Use of Laboratory Animals published by the US National Institutes of Health. Appropriate measures were taken to minimize the number and suffering of animals.

### 2.2. Chronic Constriction Injury (CCI Rat Model and Drug Administration

A model of CCI was induced in rats using a previously described method [23], with 8 rats in each group. Following anesthesia via intraperitoneal injection of sodium pentobarbital (50 mg/kg), rats were placed in a lateral decubitus position on the nonoperated side. An incision was performed at the mid‐thigh of the ipsilateral hindlimb. The underlying muscles were subjected to blunt dissection to expose the right sciatic nerve. Under microscopic visualization, 4 ligatures of 4‐0 chromic gut were tied around the sciatic nerve at 1‐mm intervals. The tightness was carefully adjusted to produce a slight constriction of the nerve diameter without interrupting the epineural blood circulation. The incision was closed in layers and disinfected with povidone–iodine. Postoperatively, all rats were housed individually. Sham controls received identical surgical exposure without ligation. After a 12‐h recovery period, esketamine (5 mg/kg) [24, 25] was administered intraperitoneally, once daily for a week. Sham and CCI groups were given an equal volume of physiological saline via the same route.

### 2.3. Viral Packaging

Lentiviral vectors were constructed by cloning specific oligonucleotide sequences into the pU6‐Luc‐Puro backbone (Hanbio, LV124). These sequences included those for miR‐153–3p mimic (miR‐mimic) and miR‐153–3p inhibitor (miR‐inhibitor) with negative control (miR‐NC), AKT3‐specific small interfering RNA (si‐AKT3), and corresponding negative control (si‐NC). Lentiviral vectors were administered into the intrathecal space (L4–L6) through a catheter. These genetic interventions were performed 3 days before CCI. L4–L6 dorsal spinal cord segments were isolated for quantitative protein and RNA assessment.

### 2.4. Paw Withdrawal Mechanical Threshold (PWMT)

Before the PWMT assay, rats were given a 30‐min acclimatization period on the metal mesh floor. PWMT referred to the minimum force of von Frey filaments required to elicit a rapid withdrawal reflex upon perpendicular stimulation of the plantar surface. Each filament was applied five times, and the threshold was determined as the lowest force inducing a positive response in at least three out of five trials.

### 2.5. Paw Withdrawal Thermal Latency (PWTL)

The thermal sensitivity of CCI rats was determined using the Hargreaves apparatus. Results were expressed as the PWTL. To prevent thermal tissue injury, a cutoff time of 18 s was established. All experiments were conducted in a quiet environment to minimize external disturbances affecting rat behavior.

### 2.6. RT‐qPCR

The RNA isolation was performed by the Trizol (Invitrogen, 15,596–018) method. Following the specifications, cDNA was obtained by the PrimeScript RT system (Takara, RRO36A). To assess the transcriptional abundance via qPCR (Takara, RR820A), U6 and GAPDH were employed as references to normalize miR‐153–3p and AKT3, respectively, with the 2^−ΔΔCT^ approach.

### 2.7. ELISA Assay

The protein concentrations of proinflammatory cytokines (IL‐6, TNF‐α, and IL‐1β) in spinal cord homogenates were measured by specific commercially available ELISA kits (Elabscience, E‐EL‐R0015, E‐EL‐R0019, and EL‐R001). The supernatants of tissue homogenates were prepared by centrifugation at 4°C. After specific antigen–antibody binding, enzyme‐labeled detection antibodies were added to capture the immune complexes. Absorbance from the colorimetric substrate conversion was measured at 450 nm on a microplate reader.

### 2.8. Western Blot

Protein samples were lysed in RIPA buffer (Thermo Scientific, 89,900), quantified via BCA assay, and mixed with SDS‐PAGE loading buffer, followed by 10‐min boiling. After separation on 10% SDS‐PAGE gels (100 V) and transferring to PVDF membranes, the membranes were blocked and probed with primary antibodies overnight at 4°C. Blocking solution was used to dilute specific primary antibodies of AKT3 (CST,4059), *p*‐IKKα/β (Santa Cruz, sc‐23470‐R), p‐p65 (CST, 3033), p65 (Proteintech, 10745‐1‐AP), iNOS (CST, 13,120), COX2 (CST, 4842), and *β*‐actin (Abcam, ab8226). Following TBST washing, membranes were incubated with secondary antibodies for 1.5 h at room temperature and then visualized using chemiluminescence detection.

### 2.9. Bioinformatics Analysis and Target Prediction

Online databases (TargetScan, miRDB, and ENCORI) were employed for target screening. Results are shown in the Venn diagram. The common elements from this diagram were then mapped to KEGG pathways using the DAVID bioinformatics resource. Significantly enriched pathways were identified via a hypergeometric test with a significance threshold of *p* < 0.05. Bubble plots were generated to visualize the enrichment factor and gene count for each pathway.

### 2.10. Dual‐Luciferase Reporter Analysis

Wild‐type and mutant AKT3 3′UTR sequences were cloned into pmirGL3 (Promega, E1500) to generate luciferase reporter constructs. They were co‐transfected with miR‐153–3p mimic (miR‐mimic), miR‐153–3p inhibitor (miR‐inhibitor), or negative controls (miR‐NC). Forty‐eight hours after transfection, luciferase activity was quantified using a luminometer to assess miR‐153–3p‐mediated regulation of AKT3 expression.

### 2.11. RIP Analysis

Cell lysates were co‐incubated overnight at 4°C with magnetic beads coupled with anti‐Ago2 or anti‐IgG antibodies according to the Magna RIP kit (Millipore, 17–700). After incubation, the bead complexes were rigorously washed to eliminate background noise. RNA was extracted from the complexes, and the abundance of miR‐153–3p and AKT3 was measured via RT‐qPCR to calculate their fold enrichment.

### 2.12. Statistical Analysis

Data were analyzed by GraphPad Prism 7 and shown as mean ± SD. To evaluate intergroup variations, Student’s *t*‐test, one‐way ANOVA, or two‐way ANOVA with Tukey’s post hoc correction was used to identify significant differences (*p* < 0.05).

## 3. Results

### 3.1. Esketamine’s Protective Role in CCI Rats

**FIGURE 1:**
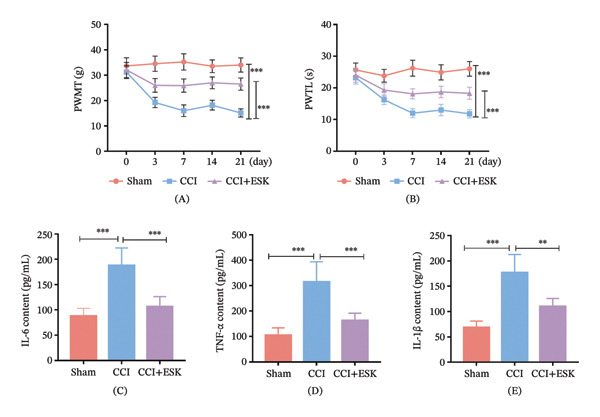
Esketamine’s protective role in CCI rats. (A) PWMT. (B) PWTL. (C) Content of IL‐6. (D) Content of TNF‐α. (E) Content of IL‐1β. ^∗∗^ *p* < 0.01 and ^∗∗∗^ *p* < 0.001.

Behavioral testing showed that CCI treatment significantly reduced PWMT and PWTL relative to the sham group, while these changes were alleviated by esketamine treatment (Figures 1A and B). Profiling of neuroinflammation‐related cytokines indicated a pronounced increase in the expression of IL‐6 (Figure 1C), TNF‐α (Figure 1D), and IL‐1β (Figure 1E). Subsequent esketamine administration attenuated the levels of the above cytokines. Thus, esketamine conferred protective effects against neuropathic pain in CCI rats.

### 3.2. Regulation of miR‐153–3p Expression by Esketamine in CCI Rats

**FIGURE 2:**
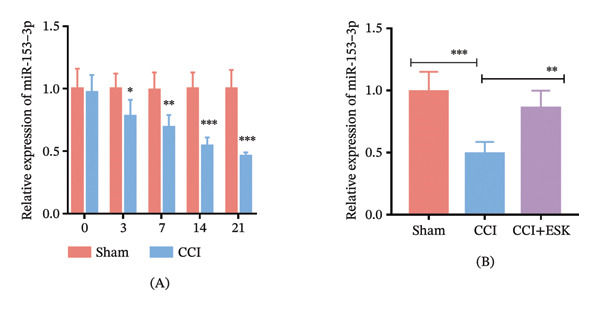
Regulation of miR‐153–3p expression by esketamine in CCI rats. (A) Time course of miR‐153–3p expression in the spinal cord after CCI. (B) Effect of esketamine administration on miR‐153–3p levels. ^∗^ *p* < 0.05, ^∗∗^ *p* < 0.01, and ^∗∗∗^ *p* < 0.001.

We then investigated whether esketamine affected the expression of miR‐153–3p. First, we measured miR‐153–3p levels in the spinal cord at multiple time points postsurgery. As shown in Figure 2A, compared to the sham‐operated group, miR‐153–3p expression was significantly reduced in CCI rats. Notably, administration of esketamine effectively reversed this downward trend (Figure 2B). These results indicated that in the CCI model, esketamine upregulated the expression of miR‐153–3p.

### 3.3. Functional Validation of miR‐153–3p Upregulation in CCI Rats

**FIGURE 3:**
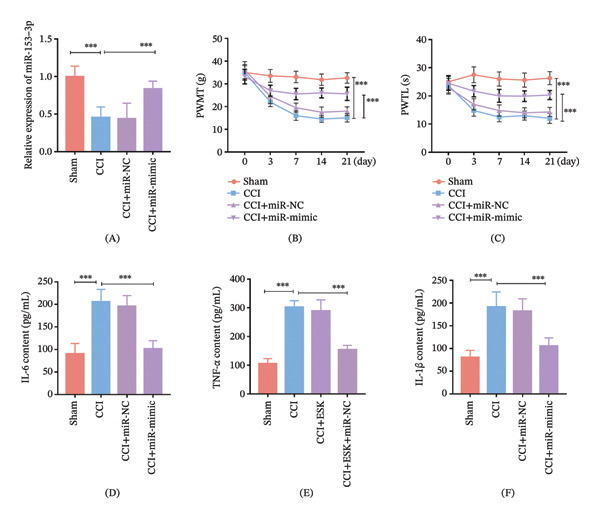
Functional validation of miR‐153–3p upregulation in CCI rats. (A) Relative expression of miR‐153–3p after mimic delivery. (B) Effect of miR‐153–3p upregulation on PWMT. (C) Effect of miR‐153–3p upregulation on PWTL. (D–F) Effect of miR‐153–3p upregulation on IL‐6, TNF‐α, and IL‐1β. ^∗∗^ *p* < 0.01 and ^∗∗∗^ *p* < 0.001.

To investigate the functional role of miR‐153–3p, we delivered miR‐153–3p mimic into CCI rats. The decrease in miR‐153–3p was effectively abrogated by mimic delivery (Figure 3A). The significantly elevated pain perception, as indicated by decreased PWMT and PWTL, was attenuated by the miR‐153–3p mimic (Figures 3B and C). In addition, the increased neuroinflammation‐related cytokines were reversed by miR‐153–3p upregulation (Figures 3D–F). Thus, elevating miR‐153–3p mimics the protective effect of esketamine.

### 3.4. Downstream Target of miR‐153–3p

Target prediction identified 240 common genes (Figure 4A). KEGG analysis revealed that the elements of the neurotrophic signaling pathway (marked with a rectangle) included 10 genes (GRB2, FOXO3, PIK3R1, SHC1, AKT3, BCL2, GAB1, MAP3K1, RPS6KA5, and SH2B3), as shown in Figure 4B. They were further validated by RIP and dual‐luciferase reporter analysis. RIP assay confirmed that both miR‐153–3p and AKT3 were apparently enriched in the anti‐Ago2 group (Figure 4C). In addition, miR‐153–3p mimic reduced the luciferase activity of WT‐AKT3, while its inhibitor enhanced the luciferase activity. Nevertheless, the luciferase activity of MUT‐AKT3 was unaffected by miR‐153–3p levels (Figure 4D). Detection of AKT3 levels at different time points confirmed a significant elevation in CCI rats (Figure 4E). This elevation was then reversed by subsequent AKT3 knockdown (Figure 4F). Functional assessment showed that AKT3 knockdown largely restored pain sensitivity, evidenced by increased PWMT and PWTL values (Figures 4G and H). Assessment of inflammatory cytokines revealed that AKT3 knockdown significantly counteracted their aberrant elevation observed in CCI rats (Figures 4I–K).

FIGURE 4Downstream target of miR‐153–3p. (A) Target prediction of miR‐153–3p in a Venn diagram. (B) Signal pathway analysis with common genes. (C) RIP assay for miR‐153–3p and WT‐AKT3. (D) Luciferase activity assay based on binding sites. (E, F) Relative mRNA level of AKT3. (G) Effect of AKT3 knockdown on PWMT. (H) Effect of AKT3 knockdown on PWTL. (I–K) Effect of AKT3 knockdown on IL‐6, TNF‐α, and IL‐1β. ^∗∗^ *p* < 0.01 and ^∗∗∗^ *p* < 0.001.

**Figure:**
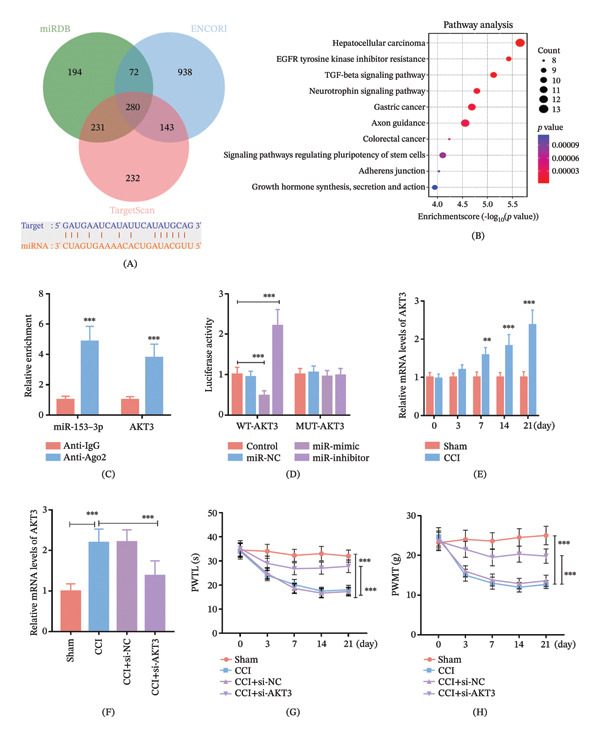

**Figure:**
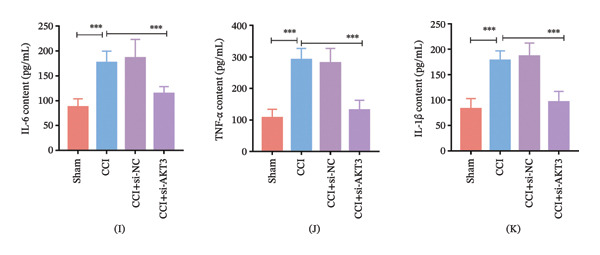

### 3.5. Reversal of miR‐153–3p Inhibition on Esketamine Protection by AKT3 Knockdown

In rescue experiments, esketamine treatment reversed the expression of both miR‐153–3p (Figure 2B) and AKT3 (Figure 5A). AKT3 was upregulated following miR‐153–3p inhibition in esketamine‐treated CCI rats, which was effectively reversed by AKT3 knockdown (Figure 5B). Functional validation confirmed that decreased PWMT and PWTL were then reversed by AKT3 knockdown (Figures 5C and D). Although miR‐153–3p inhibition significantly enhanced inflammatory cytokines, these increases were effectively counteracted by AKT3 knockdown (Figures 5E–G).

FIGURE 5Reversal of miR‐153–3p inhibition on esketamine protection by AKT3 knockdown in CCI rats treated with esketamine. (A) Relative mRNA levels of AKT3 in the CCI model after esketamine administration. (B) Relative expression of AKT3. Detection on PWMT (C) and PWTL (D). Detection of proinflammatory cytokines, IL‐6 (E), TNF‐α (F), and IL‐1β (G). ^∗∗^ *p* < 0.01 and ^∗∗∗^ *p* < 0.001.

**Figure:**
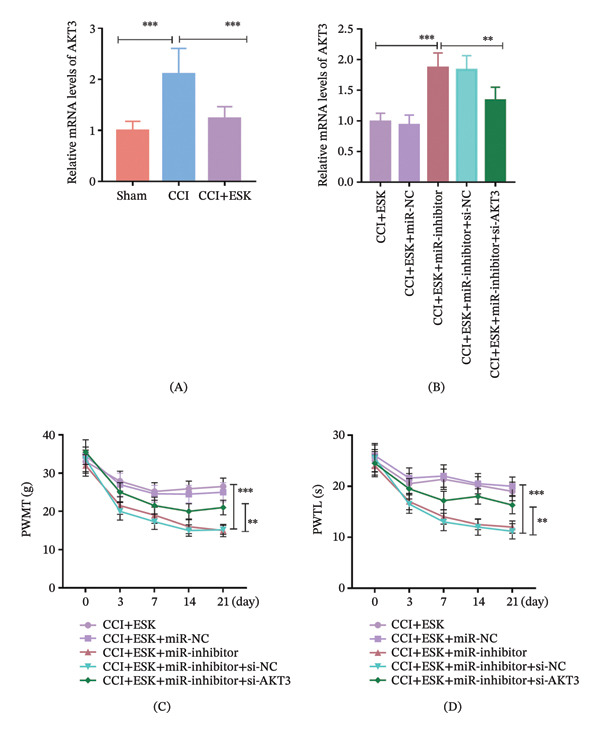

**Figure:**
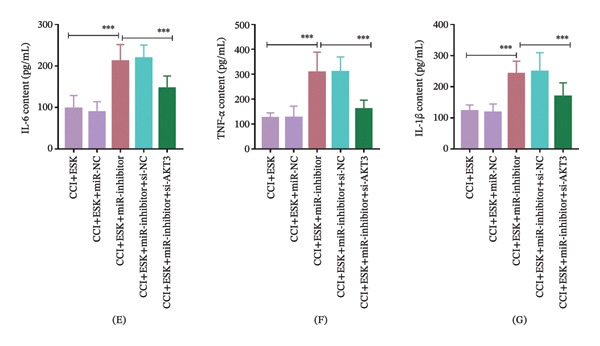

**FIGURE 6:**
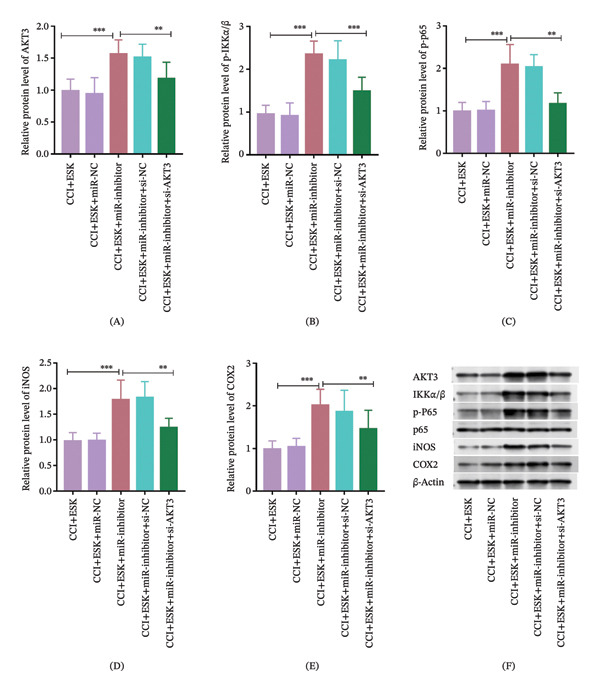
AKT3/NF‐κB cascade triggered by miR‐153–3p inhibition. The spinal cord protein levels of (A) AKT3, (B) *p*‐IKKα/β, (C) p‐p65, (D) iNOS, and (E) COX2 were measured by (F) Western blot in CCI rats. ^∗∗^ *p* < 0.01 and ^∗∗∗^ *p* < 0.001.

To explore the mechanism, AKT3/NF‐κB signaling, and downstream effectors, the AKT3/NF‐κB pathway and its downstream effectors were further assessed to clarify the underlying mechanism. In esketamine‐treated CCI rats, miR‐153–3p inhibition elevated protein levels of AKT3 (Figure 6A), *p*‐IKKα/β (Figure 6B), p‐p65 (Figure 6C), iNOS (Figure 6D), and COX2 (Figure 6E). Notably, AKT3 knockdown effectively abolished their elevation. The above protein bands are presented in Figure 6F. Thus, miR‐153–3p′s neuroprotective effect was at least partially mediated by the NF‐κB signaling pathway.

## 4. Discussion

In the CCI‐induced neuropathic pain model, our findings revealed that esketamine effectively alleviated pain hypersensitivity and suppressed the release of spinal proinflammatory cytokines. Mechanistically, these neuroprotective outcomes were mediated via modulation of the miR‐153–3p/AKT3 signaling axis.

Behavioral data from our study demonstrated esketamine’s efficacy in alleviating CCI‐associated pain sensitivity, as evidenced by elevated PWMT and PWTL. These results align with accumulating evidence from recent investigations. In the context of open abdominal surgical procedures for gynecological cancers, esketamine has emerged as a reliable postoperative analgesic agent, ensuring both safety and efficacy [26]. Another clinical application confirmed its superior analgesic effect compared to sufentanil [27]. While adverse effects warrant attention as reported in recent literature [28], esketamine exhibits a favorable safety profile, with minimal respiratory depression in the safe dosage range [29]. In addition, compared to opioids, esketamine can reduce postoperative delirium risk [30] and promote gastrointestinal function recovery [31] in therapeutic settings. Besides its role in mitigating surgical pain, our findings expanded its therapeutic potential in modulating neuropathic pain.

MiR‐153–3p was confirmed as a central mediator in esketamine‐induced neuroprotection. We found miR‐153–3p was significantly reduced in CCI rats, and esketamine administration effectively reversed its decline. Functional validation showed that miR‐153–3p upregulation was sufficient to suppress pain hypersensitivity and proinflammatory cytokines, exhibiting similar effects to esketamine. Increased miR‐153–3p has been established to exert a critical protective role in various neurological disorders, including suppression of microglial inflammatory response [18] and reduction of hippocampal neuronal apoptosis [32]. Our findings supported that miR‐153–3p served as a central molecular effector underlying esketamine’s analgesic effect.

AKT3 was identified as a downstream target of miR‐153–3p. The increased pain sensitivity induced by miR‐153–3p inhibition was attenuated by AKT3 downregulation. The negative correlation between miR‐153–3p and AKT3 expression levels has been shown in previous reports [33]. Results from a CCI‐induced animal model confirm that AKT3 expression in the spinal cord is significantly upregulated, which is closely correlated with exacerbated neuroinflammation [34]. Furthermore, research has demonstrated that upregulation of AKT3 represents a pivotal molecular mechanism connecting nerve injury and neuroinflammation to pain behavior [35]. Thus, the miR‐153–3p/AKT3 axis was a critical molecular pathway in esketamine’s neuroprotective action.

Inflammation drives neuropathic pain onset and progression through immune cell activation and massive proinflammatory cytokine release [36]. Following nerve injury, microglia‐derived proinflammatory mediators, such as IL‐1β, IL‐6, and TNF‐α, directly act on neurons to enhance their excitability and amplify pain perception [37]. As central effectors of neuroinflammation, iNOS and COX‐2 drive neuronal injury, heightened oxidative stress, and synaptic dysfunction through the overproduction of prostaglandins, nitric oxide, and other inflammatory mediators [38]. Here, we demonstrated that miR‐153–3p suppression significantly increased COX‐2 and iNOS expression alongside NF‐κB pathway activation. Simultaneous AKT3 knockdown abrogated this proinflammatory phenotype. This suggested that the miR‐153–3p/AKT3 axis was a pivotal modulator of inflammation, acting at least partially through the NF‐κB signaling pathway.

Limitations can also be observed in this study. While this approach avoids interference from the estrous cycle on the experimental results, it limits the generalizability of the conclusions to female populations. Future studies should further investigate whether the analgesic effects and inflammatory regulatory roles of the esketamine/miR‐153–3p/AKT3 pathway differ between male and female animals. In addition, our findings are derived from animal models, but the underlying molecular mechanisms remain to be further validated and refined in cellular models. Therefore, in addition to validation in female animals, in vitro verification is also required in microglia, neurons, astrocytes, and their co‐culture systems.

Collectively, we found that esketamine exerted analgesic effects in neuropathic pain and concurrently dampened inflammatory cytokine release through the miR‐153–3p/AKT3 pathway. This provided insights for optimizing the clinical application of esketamine in neuropathic pain management. In this study, we use only adult male SD rats and do not include female animals.

## Funding

No funding was received to assist with the preparation of this manuscript.

## Ethics Statement

This experiment was conducted with the approval of the Animal Ethics Committee of the GuannanCounty First People’s Hospital. All animal experiments in this study were in strict accordance with the protocols stated in th*e* Guide for the Care and Use of Laboratory Animals published by the US National Institutes of Health.

## Consent

Appropriate measures were taken to minimize the number and suffering of animals.

## Conflicts of Interest

The authors declare no conflicts of interest.

## Data Availability

The data that support the findings of this study are available from the corresponding author upon reasonable request.
